# A Mini-Review of Relationships Between Cannabis Use and Neural Foundations of Reward Processing, Inhibitory Control and Working Memory

**DOI:** 10.3389/fpsyt.2021.657371

**Published:** 2021-04-22

**Authors:** Kristen P. Morie, Marc N. Potenza

**Affiliations:** ^1^Department of Psychiatry, Yale University School of Medicine, New Haven, CT, United States; ^2^Child Study Center, Yale University School of Medicine, New Haven, CT, United States; ^3^Connecticut Mental Health Center, New Haven, CT, United States; ^4^Connecticut Council on Problem Gambling, Wethersfield, CT, United States; ^5^Department of Neuroscience, Yale University School of Medicine, New Haven, CT, United States

**Keywords:** sustance-related disorders, addictive behaviors, cannabis, cannabidiol, cognition, reward, impulsiveness

## Abstract

Cannabis is commonly used, and use may be increasing in the setting of increasing legalization and social acceptance. The scope of the effects of cannabis products, including varieties with higher or lower levels of Δ9-tetrahydrocannabinol (THC) or cannabidiol (CBD), on domains related to addictive behavior deserves attention, particularly as legalization continues. Cannabis use may impact neural underpinnings of cognitive functions linked to propensities to engage in addictive behaviors. Here we consider these neurocognitive processes within the framework of the dual-process model of addictions. In this mini-review, we describe data on the relationships between two main constituents of cannabis (THC and CBD) and neural correlates of reward processing, inhibitory control and working memory.

## Introduction

Cannabis is widely used. The 2018 Monitoring the Future survey indicated that approximately one-fifth of adolescents had tried cannabis by 12th grade (1), with frequencies of past-month use having increased over several years (2). There has been increasing legalization of cannabis and cannabis-derived products (3), and a commensurate increase in novel ways to consume these products, including edibles, pills and vaping (4–6). Novel routes of consumption have accompanied products with varying amounts of Δ9-tetrahydrocannabinol (THC) and cannabidiol (CBD), including ones that contain only CBD, such as oils or gummies. Table 1 illustrates several of these products, and it is likely that usage rates and formulations will continue to change as new products are developed.

**Table 1:** Examples of methods of cannabis administration.

	**Combustible**	**Edibles**	**Vape/dab**
Product/method	Smoking joints, pipes	Gummies, capsules, pills, cannabis-infused food and drink	Oils, shatter/butter
THC or CBD content	Chemovar Type I THC >0.3% and CBD <0.5%, THC dominant	Grams of THC range 1.2–5 mg (microdose) to 10 mg (recreational dose with low tolerance)	Oils up to 75% THC, 0.2% CBD (rest is non-THC content such as flavors and pigments)
	Chemovar Type II approximate 1:1 ratio THC/CBD	40–50 mg THC per day (medical grade pain relief) to 100 mg (recreational users with high tolerance)	Shatter/butter up to 80–90% THC
	Chemovar Type III <0.3% THC, CBD-Dominant	Products also include CBD only with essentially no THC (derived from Chemovar type III)	CBD oils and CBD shatter (derived from Chemovar Type III)

Increases in legalization and multiple consumption methods have accompanied changes in perceptions, with more individuals perceiving marijuana products as safe and non-addictive (7, 8). However, individuals with heavier use of cannabis and cannabis use disorder (CUD) typically report lower qualities of life (9). Longer-term ramifications of use of different cannabis products, specifically on neural and cognitive processes associated with engagement in addictive behaviors, remain understudied. As increasing legalization looms and use of cannabis products becomes increasingly socially acceptable, understanding potential effects of cannabis use on the brain, and how alternate methods of use or different cannabinoid products may affect the brain and propensities for addictive engagement, is particularly important.

Recent reviews of cannabis use have focused on epidemiological considerations and how use patterns have changed as legalization continues (10), and the ramifications of cannabis use on multiple domains examined using fMRI (11). Differences between THC and CBD have also been reviewed, with a focus on how acute administration may effect blood flow and neural activation (12). Here, we review data specifically relevant to the dual-process model of addiction on how cannabis may impact domains associated with reward processing and inhibitory control, as well as working memory. Each of these domains has been linked to addictive behaviors (13, 14). We review select preclinical, behavioral and brain imaging research using functional magnetic resonance imaging (fMRI) and additionally consider electroencephalography (EEG), which has not been included in past imaging-centered reviews of effects of cannabis. We also discuss differences between THC and CBD, which have very different effects.

## THC and CBD

Cannabis contains multiple cannabinoids, and the two that have received most research attention are THC (15) and CBD (16). THC is a psychoactive compound, with neurotropic effects including “highs” (17), anxiety (18), and psychosis (19, 20), the risk for which is increased with higher quantities of THC consumed (21). CBD acts as an indirect antagonist of THC's effects (16). CBD binds less tightly than THC to CB1 and CB2 receptors, and, while acute administration of THC often results in anxiety, dysphoria, and increased heart rate, effects of acute administration of CBD and placebo on these measures were indistinguishable, not generating significant changes (22). CBD is a negative allosteric modulator of the CB1 receptor (23), modifying the receptor's affinity for THC and potentially reducing THC's effects (24). Brief explanations for the mechanisms of action for THC and CBD and their binding potential are illustrated in Table 2, although it should be mentioned that binding affinities for these substances do not always correspond to their effects on cell action (29). CBD products, such as oils or tinctures, are typically derived from the “hemp” strain of the cannabis plant (Chemovar type III), which contains 0.3% or less THC by weight, while THC products are typically derived from high THC strains (Chemovar type I). There is little evidence of CBD alone having strong abuse liability (30–32). Despite the burgeoning use of cannabis-derived oils, tinctures and edibles in specific forms or with specific formulations focused upon THC or CBD, investigations of specific cannabinoids on domains of working memory, reward processing and inhibitory control are relatively scarce.

**Table 2:** Cannabis pharmacology—THC and CBD.

**THC pharmacology**	**CBD pharmacology**
Partial agonist of CB1 receptors, 5HT3 receptors in CNS -> inhibition of the release of acetylcholine and glutamate -> influencing y-aminobutyric acid, N-methyl-D-aspartate, opioid and serotonin receptors.	Lowers agonist efficacy of THC by modulating CB1 receptors, binds to distinct site on CB1 receptor
Ki values 5 (25) to 50 (26)	Ki values 4,300 (27) to 4,700 (28)

*Ki values: measure of receptor affinity (high ki value = low affinity)*.

## Inhibitory Control, Reward Processing, Working Memory and the Dual Process Model of Addiction

The dual-process model of addiction suggests that sensitization of reward circuitry is coupled with poorer top-down control of reward systems, resulting in poorly controlled behaviors and drug use (33). Top-down control reflects executive functions, such as inhibitory control and working memory. Poor inhibitory control and working memory coupled with increased reward motivation may reflect imbalances in maturational trajectories of reward-related regions (34), such as the striatum, and regions involved in reward-related impulse regulation, like the prefrontal cortex (PFC), both of which are implicated in addictive disorders (14, 35). Effects on cognition may further increase risk for engagement in addictive behaviors (36), and potential effects of cannabis on these areas of brain functioning may be reflected in the “gateway drug” hypothesis wherein marijuana precedes and predisposes to other illicit drug use (37). How cannabis use may influence domains of reward processing, inhibitory control and cognitive functioning has typically focused on combustible cannabis. Alternative methods of use, including vaping and edibles, have been less well studied. Understanding effects of cannabis use, and additionally the potential effects of chronic use of THC or CBD concentrates, is particularly important given ongoing legalization efforts.

## Cannabis and Working Memory

Early investigations of cognition, particularly working memory, have indicated that acute cannabis use is associated with impairments in holding, manipulating and remembering information (38–40), with impairments typically remaining after other acute effects have subsided. Memory deficits are apparent in cannabis-using college students after 24 h of abstinence (41) and with heavy use (42), and these deficits are associated with duration of use (43, 44). Imaging has revealed altered activation during working memory tasks in regions such as the anterior cingulate and the thalamus even after sustained abstinence, both in adults (45, 46) and adolescents (47). However, some data suggest that working memory impairments may precede cannabis use. In a 3-year examination of individuals with heavy cannabis use, no changes in working-memory-related brain activations (in the bilateral frontal poles and ventrolateral prefrontal, dorsolateral prefrontal, premotor, paracingulate, and inferior parietal cortices) were observed over time (48). Activation during an N-Back working memory task did not differ between individuals with and without cannabis use; however, greater activation statistically predicted escalation of cannabis use (49). While the weight of the literature points to working-memory impairments associated with cannabis use, preexisting vulnerabilities in working memory may exist and contribute to heavy use.

## THC and CBD and Working Memory

THC has been proposed to be the primary culprit in working-memory impairment associated with cannabis use. This has been demonstrated in animal models, where exposure to THC during adolescence resulted in learning impairments (50) that persisted into adulthood (51, 52). Acute examinations of THC in humans also suggest robust effects on memory. In a study where several memory tasks were administered to adults who were given acute oral THC, THC produced increased error rates alongside faster performance (53). Similarly, acute THC administration in healthy adults impaired performance on the Wisconsin Card Sorting Task (54). However, in both studies, performance returned to normal once effects of THC had subsided. Other work has examined neural correlates of attention and working memory in individuals given intravenous THC, where it was found that the P300 amplitude, related to responses to novel stimuli, was reduced and the level of reduction correlated with subjective reports of altered perceptions (55).

In contrast, CBD may enhance cognition, particularly in cannabis-using populations (56), schizophrenia (57–59) and neurodegenerative diseases (60, 61). CBD may reduce cognitive decrements seen in people who smoke cannabis (24). An animal study demonstrated that CBD improved memory among cognitively impaired rats (62). However, no effects were seen in rats who were not impaired. In humans, effects of acute use of vaped CBD and THC on attention or simulated driving may not differ between substances (63). Further, among abstinent individuals who smoke tobacco, acute CBD administration impaired working memory and increased errors of commission during N-back task performance (64). While evidence suggests that CBD may have promise for alleviating cognitive impairment in cannabis-using or clinical samples (16, 65), more research is needed on how it may influence working memory in other populations.

## Cannabis and Inhibitory Control

Response inhibition and behavioral control, including over drug-seeking, is important in addictive disorders (66). Impairments in inhibitory control may promote risky or disadvantageous decision-making in people who use cannabis (67). Poor inhibitory control during a Go/No-Go task and disadvantageous decision-making during a gambling task have been observed in cannabis-using young adults (68), consistent with findings among general adults (69). Differences in neural correlates of inhibitory control associated with cannabis use do not appear entirely consistent. Regions associated with inhibitory control show altered activation in people who use cannabis, with lower prefrontal activation as measured by fMRI, consistent with findings in alcohol and stimulant use disorders (14). During a Go/No-Go task in cannabis-using vs. non-cannabis-using adults, the former vs. latter group showed no differences in commission errors, but showed reduced error monitoring that was associated with reduction in activation of the anterior cingulate and right insula (70). Functional imaging during a Stop-Signal Task also revealed no differences in an inhibitory network activation between cannabis-using vs. non-cannabis-using individuals, but revealed that the former group had greater connectivity between a right frontal control network and substantia nigra/subthalamic nucleus network when functional connectivity was examined (71). In a study employing a Go/No-Go task in adolescents who were abstinent for two weeks, greater BOLD responses were observed in the left frontal cortex, left cingulate cortex, and the left thalamus during correct response inhibitions in those who used cannabis, though this may reflect greater inhibitory effort required to remain abstinent (72). EEG has revealed inhibition differences associated with cannabis use, with a reduction in the No-Go-related P3 component (a component associated with inhibitory control) of the event-related potential (ERP) when compared to non-drug-using or tobacco-using groups (73). Acute administration of cannabis before a Go/No-Go task also revealed a reduction in the No-Go P3 (74). While alterations in inhibitory control and its neural correlates appear linked to cannabis use, future work should continue examining this domain to specify precise relationships.

## THC and CBD and Inhibitory Control

An animal model that investigated impulsivity using the 5-choice serial-reaction-time test demonstrated that THC exposure resulted in increased motor impulsivity in rats that persisted after exposure ceased (75). An investigation of acute THC in humans revealed reduced activations in left inferior frontal regions that were associated with increased inhibition errors, impaired inhibition efficiency and transient psychotic symptoms (76). Acute effects of THC were also seen on an ERP associated with inhibition, the P300, and this reduction in P300 amplitude was not reversed by CBD (77). Further, an imaging study that investigated response inhibition after acute administration of either CBD or THC to healthy subjects revealed that while there were no performance differences between conditions, THC attenuated activation in the right inferior frontal and anterior cingulate gyri, regions associated with response inhibition. In contrast, CBD administration was associated with deactivation of the left temporal cortex and insula, demonstrating that CBD effects different regions, ones less typically associated with inhibition (78). Among people using CBD for treatment-resistant epilepsy, CBD altered connectivity patterns during an attentional-control task (79). It is possible that heterogeneity in findings outlined above may relate to types of cannabis used and differing effects of THC and CBD. One study has examined this, examining functional connectivity of executive, salience, and default-mode networks during resting state (80). Individuals were given cannabis containing THC (no CBD), cannabis containing THC with CBD and placebo. Reductions in functional connectivity were seen across networks for both cannabis types, and within the salience network, cannabis with THC and no CBD reduced connectivity relative to cannabis with CBD. Further, posterior cingulate connectivity was specifically impacted by cannabis with THC and no CBD, and this effect correlated with subjective “high” sensations. This study highlights that specific chemovars of cannabis, or use of different products containing CBD, THC or both, may result in different effects on inhibitory control and cognition.

## Cannabis and Reward Processing

Deficits in motivation and reward sensitivity may be pronounced with cannabis use, with several survey-based examinations linking self-reported lack of motivation and cannabis use (81). Blunted reward responses independent of alcohol or nicotine use have been observed with cannabis use, with greater blunting associated with more severe use (82, 83). Among cannabis-using relative to non-using subjects, reduced activation in the nucleus accumbens, caudate, left putamen, right inferior and medial frontal gyrus, superior frontal gyrus, and left cingulate was observed during monetary reward anticipation, with greater activation in the putamen observed during reward outcome (84). Another study in cannabis-using adults employing the monetary incentive delay task found that those with cannabis use showed reduced activation in the left caudate and inferior frontal gyrus during rewarding feedback, and increased activation in the left caudate and bilateral inferior frontal gyrus when successfully avoiding losing money (85). In a separate study, greater ventral striatal activation was observed during losing outcomes in men with vs. without CUD (86). Relatively increased activation to rewarding outcomes was seen in the ventral striatum during reward anticipation in an independent group of cannabis-using subjects, and this activation was positively correlated with lifetime cannabis use amounts and durations (87). Cannabis-using vs. non-using individuals showed greater activation during gain trials in orbitofrontal cortex and cingulate gyrus and less activation in loss trials in orbitofrontal cortex, suggesting greater sensitivity to reward and reduced sensitivity to loss (88). However, adolescents who used cannabis only did not differ from adolescents who used tobacco only, alcohol only, cannabis+tobacco, cannabis+tobacco+alcohol, and no drugs in nucleus accumbens activation during anticipation of monetary reward or loss (89). More research is required to understand reward processing in relation to cannabis use, particularly given that cannabis and tobacco use often co-occur.

## THC and CBD and Reward Processing

Acute THC administration has been associated with blunted ventral striatal activation during reward processing (90). THC is not readily self-administered, with rat models demonstrating aversiveness (91), though adolescent rats who consume THC show impairments in predicting rewards when reaching adulthood (92). THC's effects on reward processing may underlie reward-related findings seen in individuals who smoke cannabis. CBD, however, has shown different relationships. CBD does not appear associated with addictive behaviors, and rather it may alleviate craving (93), reduce relapse potential (94), and decrease addiction severity for substance-use disorders (56), thereby reducing reinforcing effects of substances. Consistently, CBD administration to rats has resulted in less self-administration of cocaine (95) or methamphetamine (96). In humans, however, CBD administered via capsules did not change reinforcing subjective effects of smoked cannabis (97). CBD administered acutely before participants performed a monetary incentive delay task showed no differences in neural activations between CBD and placebo for either reward anticipation or reward receipt (98). Data on CBD and reward processing is thus somewhat inconsistent regarding whether or not it impacts THC's or other substance's effects on reward processing. Research on CBD's effects on reward processing is relatively scarce, especially with respect to longer-term effects on reward systems.

## Conclusions

Simultaneous reduction in top-down control, including poorer inhibition and working memory, and blunted responsivity to non-drug rewards in people who use cannabis could set the stage for poorly controlled drug-seeking, consistent with dual-process models of addiction. In addition, reward deficiency models suggest that blunted responses to non-drug rewards contribute to sensation-seeking and impulsivity, and, ultimately, to addictive behaviors (99). Similar processes may underlie cannabis- and other substance-use disorders (100). Altered reward responding may contribute to sensation-seeking while poorer inhibitory control may worsen tendencies to resist drug-seeking urges. Additionally, impaired working memory may contribute to disadvantageous decision-making, and thus increased tendencies to use cannabis. Chronic cannabis use, especially of strains/varieties high in THC, is associated with alterations in brain activation and behavior related to reward processing, working memory, and inhibitory control. It's effects on these neural correlates may provide a mechanistic explanation for why cannabis use, specifically of high-THC varieties, may lead to CUD and poorer quality of life (9). However, the potential impact of CBD on these domains appears subtle or non-existent, although more work on the effects of chronic CBD use is needed.

## Future Directions and Additional Considerations

One aspect of cognition that may be specifically relevant to individuals with CUD and may supplement the dual-process model of addiction is emotional regulation. Negative affect is associated with craving for cannabis (101), and stress induced by lab-based social tasks has elicited craving for cannabis in people with CUD (102), particularly among people with low distress tolerance (103). Many individuals report using to alleviate distress (104), and edible CBD consumption may reduce social anxiety (105). Unfortunately, imaging studies of emotional regulation in CUD are scarce, and one group has identified decreased activation in bilateral frontal regions, including precentral and middle cingulate regions, during emotional reappraisal of negative affect in individuals with vs. without cannabis use (106, 107). Future work that investigates characteristics associated with cannabis use should also focus on regulation of emotion and how THC or CBD may influence affect.

Future research should focus on how types of cannabis administration, and use of different cannabinoids, may impact cognition, reward processing and inhibitory control. Vaping of cannabis flower or cannabis concentrates (e.g., THC) may release of higher concentrations of psychoactive ingredients (108, 109). Similarly, edibles derived from concentrates may generate slower onsets of effects (110) that may lead to greater ingestion of psychoactive ingredients that may generate long-lasting effects than combustible use (4). Surveys of adolescents have identified different experiences among those who primarily smoke, vape, or consume edibles, with edible varieties described as most potent (111). Thus, investigating impacts of edibles and vaping on neural processes linked to addictive behaviors is important. Studying vaping may be particularly relevant as it has been associated with deadly illness related to use of THC oils and vitamin E acetate (112). Additionally, more study on the effects of CBD alone and in combination with THC is warranted, especially as legalization of cannabis becomes more widespread.

## Author Contributions

KPM wrote the first draft of the paper and worked with the co-author on subsequent drafts. Both authors contributed to the editorial process and have approved the final submitted version of the manuscript.

## Conflict of Interest

MP has consulted for and advised Opiant Pharmaceuticals, Idorsia Pharmaceuticals, AXA, Game Day Data and the Addiction Policy Forum; has received research support from the Mohegan Sun Casino, Connecticut Council on Problem Gambling and National Center for Responsible Gaming; has participated in surveys, mailings or telephone consultations related to drug addiction, impulse control disorders or other health topics; and has consulted for law offices and gambling entities on issues related to impulse control or addictive disorders. The remaining author declares that the research was conducted in the absence of any commercial or financial relationships that could be construed as a potential conflict of interest.
